# Opportunities and challenges of applying advanced X-ray spectroscopy to actinide and lanthanide N-donor ligand systems

**DOI:** 10.1107/S1600577521012091

**Published:** 2022-01-01

**Authors:** Tim Pruessmann, Peter Nagel, Laura Simonelli, David Batchelor, Robert Gordon, Bernd Schimmelpfennig, Michael Trumm, Tonya Vitova

**Affiliations:** a Institute for Nuclear Waste Disposal, Karlsruhe Institute of Technology, Hermann-von-Helmholtz-Platz 1, 76344 Eggenstein-Leopoldshafen, Germany; b Institute for Quantum Materials and Technologies, Karlsruhe Institute of Technology, 76021 Karlsruhe, Germany; c ALBA Synchrotron Light Facility, Cerdanyola del Vallès 08290, Spain; d Institute for Photon Science and Synchrotron Radiation, Karlsruhe Institute of Technology, Hermann-von-Helmholtz-Platz 1, 76344 Eggenstein-Leopoldshafen, Germany; e PNCSRF, APS Sector 20, Argonne, IL 60439, USA; f Moyie Institute, Burnaby, BC, Canada

**Keywords:** actinides, lanthanides, HR-XANES, CC-RIXS, X-ray Raman, partitioning

## Abstract

N-donor ligands such as *n*-Pr-BTP [2,6-bis­(5,6-di­propyl-1,2,4-triazin-3-yl)­pyridine] preferentially bind trivalent actinides (An^3+^) over trivalent lanthanides (Ln^3+^) in liquid–liquid separation. However, the chemical and physical processes responsible for this selectivity are not yet well understood. Here, an explorative comparative X-ray spectroscopy and computational (*L*_3_-edge) study for the An/Ln *L*_3_-edge and the N *K*-edge of [An/Ln(*n*-Pr-BTP)_3_](NO_3_)_3_, [Ln(*n*-Pr-BTP)_3_](CF_3_SO_3_)_3_ and [Ln(*n*-Pr-BTP)_3_](ClO_4_)_3_ complexes is presented. High-resolution X-ray absorption near-edge structure (HR-XANES) *L*_3_-edge data reveal additional features in the pre- and post-edge range of the spectra that are investigated using the quantum chemical codes *FEFF* and *FDMNES*. X-ray Raman spectroscopy studies demonstrate the applicability of this novel technique for investigations of liquid samples of partitioning systems at the N *K*-edge.

## Abstract 2

Exploring the opportunities and challenges when studying actinide and lanthanide N-donor ligand systems relevant for separation technologies from the metal and ligand point of view using X-ray spectroscopy techniques and computations.

## 1. Introduction

A way to significantly reduce the volume and the heat load of a repository after some decades and the long-term radiotoxicity of spent nuclear fuel is the so-called Partitioning and Transmutation (P&T) strategy (Magill *et al.*, 2003 ▸; OECD, 2011 ▸; Salvatores & Palmiotti, 2011 ▸; González-Romero, 2011 ▸). Here the different components are separated (partitioning) and the transuranium elements are converted to short-lived or stable nuclides (transmutation) using neutron-induced fission or capture reactions. A major step in P&T is the separation of 5*f* elements, especially the minor actinides (An), from their chemically similar 4*f* counterparts. This separation is necessary, as the lanthanide (Ln) fission products have large neutron absorption cross sections and thereby compromise transmutation efficiency in the nuclear fission process. Selective liquid–liquid extraction of An^3+^ from Ln^3+^ has been demonstrated using soft nitro­gen or sulfur-donor ligands (Madic *et al.*, 2007 ▸; Ekberg *et al.*, 2008 ▸; Kolarik, 2008 ▸; Lewis *et al.*, 2010 ▸; Harwood *et al.*, 2011 ▸; Hudson *et al.*, 2013 ▸; Panak & Geist, 2013 ▸), especially heterocyclic N-donor ligands, *e.g.* bis­triazinyl­pyridines (BTP) (Case, 1971 ▸; Kolarik *et al.*, 1999*a* ▸,*b* ▸) and bis­triazinylbi­pyridines (BTBP) (Geist *et al.*, 2006 ▸) with separation factors (SFs) for Am^3+^ over Eu^3+^ greater than 100 (SF = distribution ratio *D*_Am_/*D*_Eu_; *D*_*M*_ = [*M*]_org_/[*M*]_aq_). Additionally, the Ln elements exhibit a trend in selectivity as a function of their ionic radii (Steppert *et al.*, 2009 ▸). However, 2,6-bis­(5,6-di­propyl-1,2,4-triazin-3-yl)-pyridine (*n*-Pr-BTP) has slow extraction kinetics (Weigl *et al.*, 2006 ▸) and low chemical and radiation stability (Hill *et al.*, 2000 ▸; Rat & Hérès, 2000 ▸). Modifications like different alkyl groups (Panak & Geist, 2013 ▸; Kolarik *et al.*, 1999*b* ▸; Hudson *et al.*, 2006 ▸; Trumm, Geist *et al.*, 2011 ▸), substitution in the 4-position of the pyridine ring (Trumm, Geist *et al.*, 2011 ▸; Benay *et al.*, 2011 ▸; Trumm, Wipff *et al.*, 2011 ▸) or annulation [*e.g.* 6,6′-bis­(5,5,8,8-tetra­methyl-5,6,7,8-tetra­hydro­benzo-1,2,4-triazin-3-yl)-pyridine (CyMe4-BTP) (Hudson *et al.*, 2003 ▸; Benay *et al.*, 2010 ▸), bis-2,6-(5,6,7,8-tetra-hydro-5,9,9-tri-methyl-5,8-methano-1,2,4-benzo-triazin-3-yl)-pyridine (CA-BTP) (Trumm, Wipff *et al.*, 2011 ▸)] lead to improvements only of some properties at the cost of others. Structural changes in the lateral rings (Steppert *et al.*, 2009 ▸; Halcrow, 2005 ▸; Girnt *et al.*, 2010 ▸; Bremer, Geist *et al.*, 2012 ▸; Bremer, Ruff *et al.*, 2012 ▸; Beele *et al.*, 2013 ▸; Bremer *et al.*, 2013 ▸) can lead to drastic changes in extraction properties, *e.g.* the separation factors (SF) for Am^3+^ over Eu^3+^ change from SF_Am/Eu_ = 130 for *n*-Pr-BTP (Kolarik *et al.*, 1999*a* ▸) to SF_Am/Eu_ = 5 for 6-bis­(4-ethyl-pyridazin-1-yl)-pyridine (Et-BDP) (Beele *et al.*, 2012 ▸). The physical and chemical properties leading to strong differences in the selectivity, kinetics and stability of these related organic molecules are not yet well understood. There are numerous experimental and theoretical structural studies in the literature looking for small electronic and geometric structural variations, which might be associated with differences in bonding affinities for An compared with Ln.

This work is focused on the *n*-Pr-BTP molecule and the respective An^3+^ and Ln^3+^ complexes with the *n*-Pr-BTP ligand. We applied XANES and X-ray Raman spectroscopy experimental techniques and computations with the *FEFF* (Rehr *et al.*, 2009 ▸, 2010 ▸) and *FDMNES* (Bunău & Joly, 2009 ▸) quantum chemical codes, which are element- and bulk-sensitive probes and can be used for all Ln and An elements and N in materials in solid and liquid phase, which is not always possible with other methods. Electronic and geometric structural investigations from the ‘point of view’ of the metal and the ligand can facilitate understanding the fundamental principles of the N-donor ligands’ selectivity. We detail experimental and computational approaches pointing out their advantages and limitations. Our aim is to guide related future studies.

## 2. Methods

### 2.1. Materials

The ligand investigated in this work is 2,6-bis­(5,6-di­propyl-1,2,4-triazin-3-yl)-pyridine (*n*-Pr-BTP) (Case, 1971 ▸; Kolarik *et al.*, 1999*a* ▸,*b* ▸). For quantum chemical calculations 2,6-bis-(1,2,4-triazin-3-yl)-pyridine (H-BTP) (Case, 1971 ▸; Boucher *et al.*, 2002 ▸) has been used as an approximation for *n*-Pr-BTP. 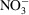 is the preferred counter ion as the extraction process of An and the separation from Ln takes place in nitric acid solution. However, the 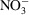 N *K*-edge spectrum has a small contribution in the *n*-Pr-BTP N *K*-edge spectrum (see Fig. 18). In 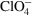 containing solution, precipitation occurs after adding *n*-Pr-BTP; therefore it is discarded as an alternative to 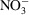 . Complexes with 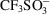 (OTf) as counter ion exhibit good solubility and OTf has a lower bonding affinity to the metal than 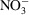 thereby enhancing the formation of 1:3 complexes. From time-resolved laser fluorescence spectroscopy (TRLFS) experiments (Panak & Geist, 2013 ▸; Trumm *et al.*, 2010 ▸) on Cm and Eu it can be seen that already above ∼0.6 mmol L^−1^ *n*-Pr-BTP concentration 1:3 complexes are formed exclusively. As an alternative, the [An/Ln(*n*-Pr-BTP)_3_](OTf)_3_ complexes were first crystallized before solving and drying on substrates. Solutions of [An/Ln(*n*-Pr-BTP)_3_](OTf/ClO_4_/NO_3_)_3_ complexes have also been dried on substrates in some of the experiments. The Ln salts that have been used in the preparation of the complexes and as reference materials for the *L*_3_-edge measurement are hydro­philic and can contain up to nine H_2_O in the first coordination shell. Especially for experiments in solution, a high H_2_O coordination has to be assumed but could not be quantified during the measurements. Calculations have been performed with [Ln(H_2_O)_9_](OTf)_3_. For the sake of simplicity, coordinating H_2_O molecules are not explicitly named throughout this work. Pu^3+^ has been shown to be stable in [Pu(*n*-Pr-BTP)_3_](NO_3_)_3_ complexes (Banik *et al.*, 2010 ▸).

### 2.2. Experiments

High-resolution X-ray absorption near-edge structure (HR-XANES) spectra and core-to-core resonant inelastic X-ray scattering (CC-RIXS) maps at Ln *L*_3_-edges were collected at the ID26 beamline at the European Synchrotron Radiation Facility (ESRF), Grenoble, France. The X-rays emitted from the sample were energy analyzed by a Johann spectrometer in scanning geometry (Glatzel *et al.*, 2009 ▸) and detected by an avalanche photodiode. The *L*_α1_ or *L*_β2_ emission lines of different Ln were diffracted and focused by spherically bent crystals with a 1 m radius of curvature at the corresponding Bragg angle [Eu *L*_α1_: Ge(333)/76.75°; Gd *L*_α1_: Si(333)/78.28°; Gd *L*_β2_: Ge(620)/77.39°].

Pu and Am *L*_3_-edge HR-XANES spectra have been collected at the INE beamline (Rothe *et al.*, 2012 ▸; Walshe *et al.*, 2014 ▸; Zimina *et al.*, 2016 ▸, 2017 ▸) at the Karlsruhe Institute of Technology (KIT) light source (KARA storage ring, KIT Campus North). The X-rays emitted from the sample were energy analyzed by a Johann spectrometer and detected by a Vortex silicon drift detector (Iwanczyk *et al.*, 2003 ▸). The *L*_α1_ emission lines of Pu (14 282 eV) and Am (14 620 eV) were diffracted and focused by five spherically bent Si(777) crystals with a 1 m radius of curvature at Bragg angle 75.7° and 71.2°, respectively. The samples were measured in solutions of 2 m*M* and 8 m*M* An concentration, respectively.

N *K*-edge XANES spectra were measured at the UE52-PGM beamline at the BESSY II synchrotron source of the Helmholtz-Zentrum Berlin using partial electron yield detection and a resolving power (*E*/Δ*E*) of ∼10000. A plane-grating monochromator (PGM) with a 1200 lines mm^−1^ grating was used to tune the incident X-rays from 380 to 420 eV. The energy of the incident X-ray beam was scanned with 0.05 eV step size. *n*-Pr-BTP, [Ho(*n*-Pr-BTP)_3_](ClO_4_)_3_ and [Ho(*n*-Pr-BTP)_3_](NO_3_)_3_ were solved in iso­propanol (10 mmol L^−1^) and ∼2 µL were dried on aluminium sample holders.

Additional N *K*-edge XANES experiments were performed in fluorescence mode at the WERA beamline at the KIT Light Source (KARA storage ring, KIT Campus North). The incident beam was monochromatized by an SGM. The energy of the incident beam was scanned with 0.05 eV step size across the pre-edge and edge region and up to 0.5 eV in the post-edge region. The scanned energy range is from 370 to 485 eV. The spectra have been calibrated using the Ni *L*_3_-edge of a NiO reference. The *n*-Pr-BTP samples were prepared by solving the ligand in ethanol and drying ∼2 µL of the solutions on Al foil.

X-ray Raman spectroscopy experiments were performed at the 20-ID beamline at the Advanced Photon Source (APS) using the Lower Energy Resolution Inelastic X-ray scattering (LERIX) spectrometer (Fister *et al.*, 2006 ▸). O *K*-edge X-ray Raman spectra of water and iso­propanol and N *K*-edge X-ray Raman spectra of crystalline *n*-Pr-BTP pressed into a pellet were measured with the analyzer energy set at 9890 eV [Si(555), θ = 88.27°]. The incident photon flux was 2 × 10^12^ photons s^−1^ using a double-crystal monochromator with Si(111) crystals. The overall experimental energy resolution was 1.3 eV estimated by measuring the FWHM of the elastically scattered radiation. The liquid samples were measured using a flow-through cell developed in cooperation with Diamond Materials GmbH (Freiburg, Germany). The cell has a diamond window with thickness of 50 µm and was set at 45° or 30° with respect to the incident beam inside a He-filled chamber. A peristaltic pump set to a flow rate of 5 mL min^−1^ was used to pump the liquids. For the crystalline sample, 18 detectors were employed and the sample was placed on a spinner to reduce radiation damage. Due to the cell blocking part of the scattered energy, only 15 detectors (cell at 30°) or 12 detectors (cell at 45°) out of 18 detectors could be used for liquid measurements.

X-ray Raman spectroscopy experiments were performed at the ID20 beamline at the ESRF using a spectrometer with 72 analyzer crystals. N *K*-edge X-ray Raman spectra of crystalline *n*-Pr-BTP and *n*-Pr-BTP solved in iso­propanol (30 mmol L^−1^) were measured with the analyzer energy set at 9690 eV [Si(660), θ = 88.5°]. The incident photon flux was ∼10^14^ photons s^−1^. The cell and pump setup was the same as used at the APS with the cell set at 45° to the incident beam. Due to the cell blocking part of the scattered X-rays only 24 detectors could be used for liquid measurements. The crystalline *n*-Pr-BTP powder was pressed into a depression in an Al plate set at 30° to the incident beam. This allowed the use of 48 crystals.

### 2.3. Computations

#### 2.3.1. Structure optimization

Ln(H-BTP)_3_ and An(H-BTP)_3_ structures were optimized imposing the *D*_3_ point group at the density functional theory (DFT) level with the BP86 functional (Becke, 1988 ▸; Perdew, 1986 ▸) employing the resolution-of-the-identity (RI) routines as implemented in *TURBOMOLE* (TURBOMOLE, 2012 ▸). Def2-TZVP basis sets (Weigend *et al.*, 1998 ▸) were used for the light elements whereas the def-TZVP basis sets (Eichkorn *et al.*, 1997 ▸) and small-core Stuttgart-PP were taken for the *f*-elements. Structures optimized in this way are denoted structure **1** throughout this work. H-BTP is used as an approximation for *n*-Pr-BTP as it has been proved that the substituents on the triazine rings hardly affect the complex structure (Berthet *et al.*, 2002 ▸; Denecke *et al.*, 2005 ▸; Petit *et al.*, 2006 ▸).

Additionally Gd(H-BTP)_3_ structures in gas and aqueous phase (called structure **2** and structure **3**, respectively) have been calculated. The structures were optimized on the DFT level in *C*_1_ symmetry using a SVP basis set and the BH-LYP functional (Becke, 1993 ▸). For the aqueous phase the structure has been solvated with COSMO-RS (Klamt, 1995 ▸).

#### 2.3.2. Calculation of Ln/An *L*_3_-edge spectra

To compare different codes and methods, the spectrum of [Eu(*n*-Pr-BTP)_3_](OTf)_3_ was calculated with *FEFF9.6* (Rehr *et al.*, 2009 ▸, 2010 ▸) and *FDMNES* (Bunău & Joly, 2009 ▸). The structural data of [Eu(*n*-Pr-BTP)_3_](OTf)_3_ were taken from unpublished X-ray diffraction data. With *FEFF*, the self-consistent field (SCF) was calculated for a cluster of 28 atoms and the full multiple scattering (FMS) for a cluster of 46 atoms. A Hedin–Lund­qvist-type exchange correlation potential was used. *FDMNES* FMS and full potential (FP) calculations were performed on a cluster of 28 atoms. In all calculations, quadrupole transitions were taken into account.

To compare different structure optimizations the experimental spectrum of [Gd(*n*-Pr-BTP)_3_](OTf)_3_ was compared with *FDMNES* FMS spectra calculated from Gd(H-BTP)_3_ optimized in the different ways described above, *i.e.* optimization with *D*_3_ symmetry, with *C*_1_ symmetry and with *C*_1_ symmetry and solvation shell.

The [Gd(*n*-Pr-BTP)_3_](OTf)_3_ spectrum and the *f* and *d* angular momentum projected density of states (*f*-, *d*-DOS) were calculated using the *ab initio* multiple-scattering theory based *FEFF9.5* code (Rehr *et al.*, 2009 ▸, 2010 ▸). The SCF and FMS calculations were performed on a cluster of 152 atoms corresponding to one [Gd(*n*-Pr-BTP)_3_](OTf)_3_ molecule. A Hedin–Lundqvist-type exchange correlation potential was used. The *f*-DOS was calculated self-consistently by using the UNFREEZEF card. The Fermi level was set to 3 eV below the calculated value of −9.6 eV in order to reproduce the pre-edge structure in the spectrum. To reach convergence, the core-hole type was set to random phase approximation (RPA card) and the core-hole potential was calculated for a cluster of 47 atoms (SCREEN card).

## 3. Results and discussion

### 3.1. Ln/An *L*_3_-edge

The electronic and geometric structures of [An/Ln(*n*-Pr-BTP)_3_](NO_3_)_3_, [Ln(*n*-Pr-BTP)_3_](CF_3_SO_3_)_3_ and [Ln(*n*-Pr-BTP)_3_](ClO_4_)_3_ complexes are probed from the metal ‘point-of-view’ using An/Ln *L*_3_-edge HR-XANES and CC-RIXS as well as HR-XANES *ab initio* quantum chemical calculations with the *FEFF* and *FDMNES* codes. Benchmark calculations using different codes and structural models are carried out.

#### 3.1.1. Ln/An *L*_3_-edge HR-XANES and CC-RIXS spectra

**Figure 1:**
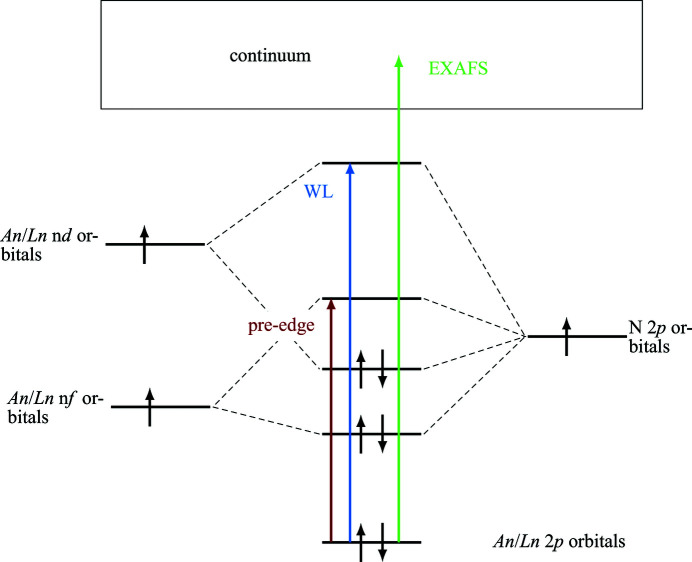
Simplified MO scheme of An/Ln bound to N and the excitations causing the pre-edge (red), WL (blue) and EXAFS (green) features observed in An/Ln *L*_3_-edge spectra.

Fig. 1 ▸ shows a simplified molecular orbital (MO) scheme of An/Ln bound to N and the excitations relevant for X-ray absorption spectroscopy (XAS) at the An/Ln *L*_3_-edge. At the white line (WL) electrons are excited from An/Ln 2*p*_3/2_ states to unoccupied molecular orbitals mixtures of An/Ln 5*d*/6*d* and N 2*p* atomic orbitals.

The WL is mainly used to observe changes in the oxidation states (Centeno *et al.*, 2000 ▸), *i.e.* the relative energy shift of the absorption edge due to the change in valence orbital occupation and the resulting change in electron density and screening of the core-hole. The WL is also sensitive to the local structure, and the density of occupied and unoccupied states, that depend also on other factors than the oxidation state, *e.g.* the chemical environment, *i.e.* type of bonding partner (Asakura *et al.*, 2014 ▸), coordination number, local symmetry (Asakura *et al.*, 2015 ▸), changes due to induced pressure (Rueff, 2009 ▸; Heathman *et al.*, 2010 ▸), *etc*. This can lead to changes in energy position, width, shape and intensity of the WL. From a quantum chemical point of view *L*_3_-edge spectra describe the angular momentum projected density of states (DOS) of unoccupied *d* and *s* (less important) like states (Mott, 1949 ▸), 5*d*/6*d* states in the case of Ln/An *L*_3_-edge spectra following the dipole selection rules Δ*l* = ±1 (Δ*J* = 0, ±1). After the first experimental observation of a weak pre-edge structure in Ce XANES (Bianconi *et al.*, 1987 ▸), band structure calculations showed a correspondence to quadrupole transitions (Finkelstein *et al.*, 1992 ▸). Even though quadrupole transitions (2*p*_3/2_ → 4/5*f*) are considerably weaker than dipole transitions (2*p*_3/2_ → 5/6*d*), they have been observed for a variety of Ln (2*p*_3/2_ → 4*f* transitions) and An (2*p*_3/2_ → 5*f* transitions) materials using X-ray emission spectrometers with an instrumental energy bandwidth similar to the core-hole lifetime broadening (Vitova *et al.*, 2010 ▸, 2013 ▸, 2015 ▸, 2018 ▸; Krisch *et al.*, 1995 ▸; Kvashnina *et al.*, 2011 ▸; Hämäläinen *et al.*, 1991 ▸; Carra & Altarelli, 1990 ▸; Tanaka *et al.*, 1994 ▸; Carra *et al.*, 1995 ▸; Bartolomé *et al.*, 1997 ▸, 1999 ▸; Gallet *et al.*, 1999 ▸; Dallera *et al.*, 2000 ▸, 2003 ▸, 2004 ▸, 2006 ▸; Journel *et al.*, 2002 ▸; Nakazawa *et al.*, 2002 ▸; Rueff *et al.*, 2004 ▸, 2006 ▸; Glatzel *et al.*, 2005 ▸; Sham *et al.*, 2005 ▸; Yamaoka *et al.*, 2006 ▸; Brouder *et al.*, 2008 ▸; Kotani, 2008 ▸). Atomic multiplet calculations of the pre-edge spectral features of Ln *L*_3_ HR-XANES demonstrated that they are shaped by electron–electron interactions and their shape, intensity and energy position on the excitation and emission energy depend on the number of available *f* electrons in the systems (Kvashnina *et al.*, 2011 ▸; Severing *et al.*, 1989 ▸; Hansmann *et al.*, 2008 ▸). The intensity of the pre-edge can also increase in the case of hybridization of Ln *f* and *d* states.

**Figure 2:**
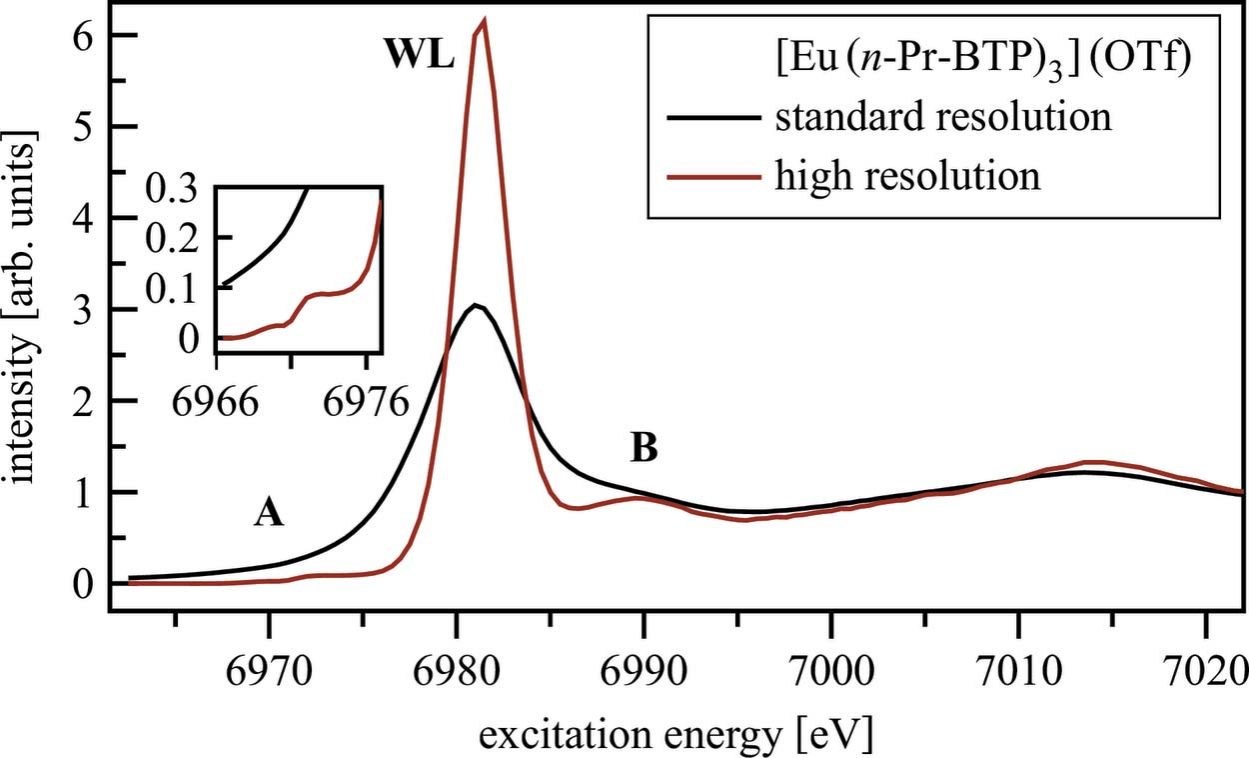
Standard and HR-XANES of [Eu(*n*-Pr-BTP)_3_](OTf)_3_.

In a standard measurement, the core-hole lifetime broadening affects the XANES spectrum leading to a significant spectral broadening compared with HR-XANES (see Fig. 2 ▸). Most notably, in HR-XANES, a pre-edge (feature A) can be resolved, the WL is sharper and has higher intensity and post-edge features (B) are well separated from the WL. The HR-XANES pre-edge and WL provide details on the electronic structure not available with conventional methods. Post-edge features close to the WL are sensitive to multiple scattering of the photoelectron with the surrounding shells and therefore the local atomic geometry around the absorbing atom, *e.g.* bonding angles, become accessible. It can complement the information relative to interatomic distances, local structural disorder, and number and kind of atoms surrounding the absorber, which can be quantitatively obtained by analyzing the extended X-ray absorption fine-structure (EXAFS) region.

**Figure 3:**
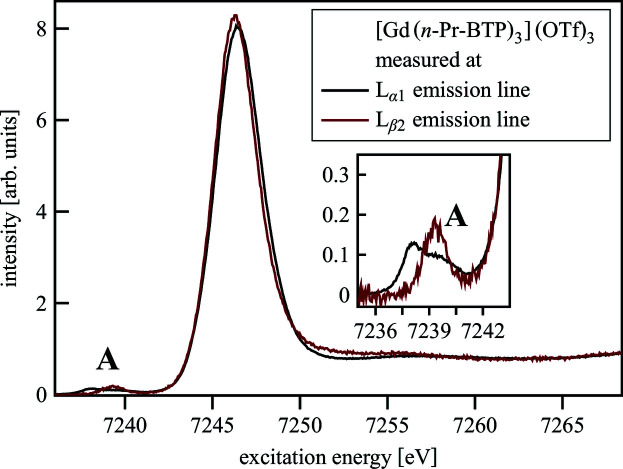
Gd *L*_3_-edge HR-XANES of [Gd(*n*-Pr-BTP)_3_](OTf)_3_ measured at the Gd *L*_α1_ and *L*_β2_ emission line.

By recording HR-XANES selecting different emission lines with similar energy resolution, it is possible to obtain HR spectra with different sensitivity, due to the different intrinsic final state core-hole lifetime broadening [*e.g.* U *L*_3_ HR-XANES collected at the *L*_β5_ emission line (Kvashnina *et al.*, 2014 ▸)]. It can also be possible to detect additional spectral features due to different screening of core-holes at different energy levels. In the case of Ln *L*_3_-edge spectra the *L*_β2_ emission line can be used instead of the conventional high-intensity *L*_α1_ emission line. However, the 4*d* core-hole resulting from the *L*_β2_ emission has a higher core-hole lifetime broadening (2 eV) (McGuire, 1972 ▸) than the 3*d* core-hole resulting from the *L*_α1_ emission (1.18 eV) (McGuire, 1974 ▸) hence no increase in resolution can be achieved. In Fig. 3 ▸ the spectra of [Gd(*n*-Pr-BTP)_3_](OTf)_3_, measured recording the emitted fluorescence by fixing the outcoming energy at the maximum of the *L*_α1_ or *L*_β2_ emission lines, are compared.

**Figure 4:**
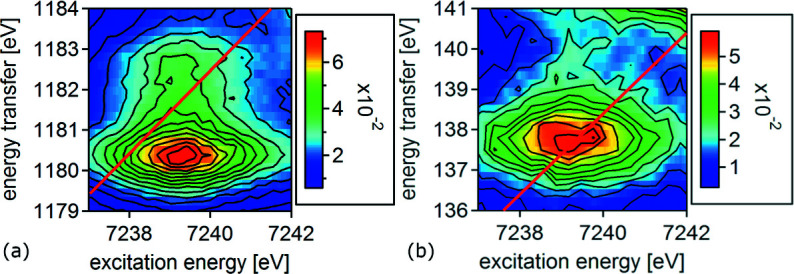
Normalized 2*p*3*d* (*a*) and 2*p*4*d* (*b*) RIXS pre-edge maps of [Gd(*n*-Pr-BTP)_3_](OTf)_3_ with XANES position (red line).

The WL of the spectrum measured at the *L*_β2_ emission line is slightly narrower and the pre-edge has only one instead of two peaks. To further investigate the differences in the pre-edge region, CC-RIXS maps were recorded. Fig. 4 ▸ shows 2*p*3*d* (*L*_α1_) and 2*p*4*d* (*L*_β2_) RIXS maps of the pre-edge of [Gd(*n*-Pr-BTP)_3_](OTf)_3_ with a red line marking the emission energy at which the HR-XANES spectra shown in Fig. 3 ▸ were measured.

The 2*p*3*d* RIXS map has two features at the same excitation energy at ∼1180.5 eV and ∼1182.5 eV energy transfer. The HR-XANES spectrum cuts through both features leading to the observed double structure in the pre-edge. In contrast, the 2*p*4*d* RIXS map has one feature at ∼137.5 eV energy transfer and additional intensity at ∼140 eV that could be the overlap of pre-edge and WL tails or a second feature comparable with 2*p*3*d* RIXS, but with lower intensity. The HR-XANES spectrum only intersects the maximum of the first feature resulting in a single pre-edge peak. Because the differences in the CC-RIXS maps appear along the energy transfer scale, they are related to final state effects. In this case these are different splitting of the 2*p* ^6^3*d* ^9^5*d* ^1^ and 2*p* ^6^4*d* ^9^5*d* ^1^ final states, respectively; changes in the screening of the core-hole cause an energy shift of the pre-edge resonance relative to the normal emission, *i.e.* the maximum of the emission line in the post-edge range. Since the 4*f*–4*f* electron–electron interactions have the same influence on both spectra, the appearance of two resonances along the energy transfer scale is likely related to differences in the 3*d*–4*f* and 4*d*–4*f* electron–electron interactions. It was shown that they have the highest influence on the spectrum after the 4*f*–4*f* electron–electron interactions (Kvashnina *et al.*, 2011 ▸).

#### 3.1.2. Calculations of Ln *L*_3_-edge HR-XANES spectra of [Ln(*n*-Pr-BTP)_3_](OTf)_3_

**Figure 5:**
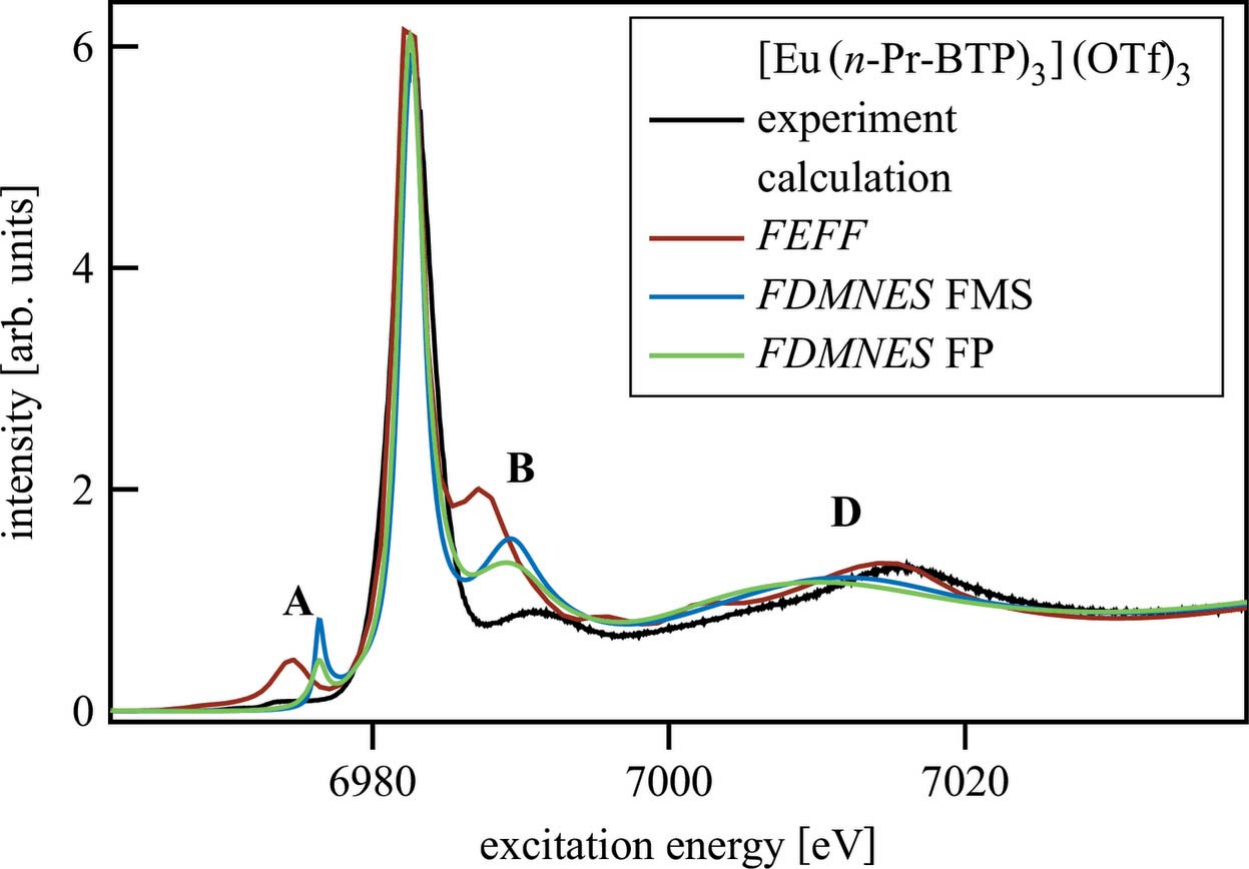
Calculations of [Eu(*n*-Pr-BTP)_3_](OTf)_3_ with *FEFF* and *FDMNES* with FMS and FP compared with the experimental spectrum.

Ln *L*_3_-edge HR-XANES spectra of Ln(H-BTP)_3_ and [Ln(*n*-Pr-BTP)_3_](OTf)_3_ were calculated with different codes and various structural models to find the best agreement between experimental and theoretical spectra. In Fig. 5 ▸ the effects of using different codes (*FEFF* and *FDMNES*) and methods (FMS and FP) to calculate an Eu *L*_3_-edge spectrum of [Eu(*n*-Pr-BTP)_3_](OTf)_3_ are shown and compared with experimental data. The calculated spectra are less broadened than the experimental spectrum to be able to see all features clearly. Therefore, the pre-edge (A) and the first post-edge feature (B) of the calculated spectra show significantly larger intensity compared with the experimental spectrum. The *FEFF* and *FDMNES* calculations reproduce well the A, D (*FEFF*) and B (*FDMNES*) features, respectively. Between the *FDMNES* FMS and FP calculations there are intensity differences for the A and B features and a small energy shift for the D peak. Since the differences are small and an FP calculation takes significantly longer than an FMS calculation (FMS: 1 h; FP: 1 week), FP calculations were not further applied.

**Figure 6:**
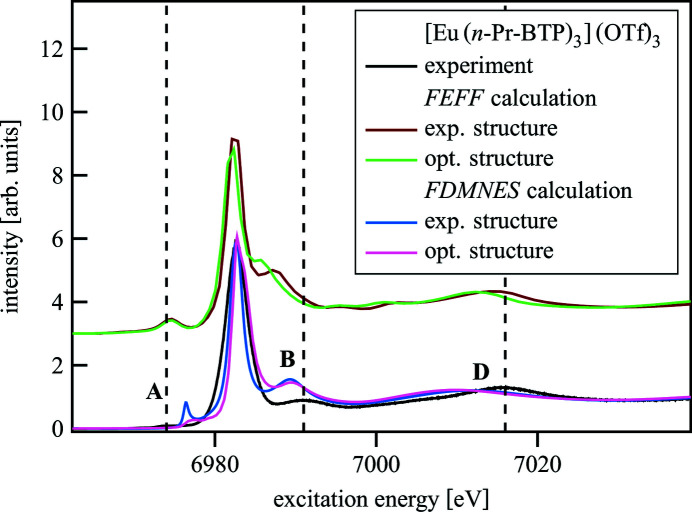
Calculations of [Eu(*n*-Pr-BTP)_3_](OTf)_3_ with *FEFF* and *FDMNES* with experimental and DFT optimized structures compared with the experimental spectrum.

Fig. 6 ▸ depicts the differences in the calculated spectra resulting from different structures used in the calculation. Both *FEFF* and *FDMNES* calculations were performed for a DFT optimized Eu(H-BTP)_3_ structure. They are compared with the *FEFF* and *FDMNES* FMS spectra shown also in Fig. 5 ▸ calculated with the experimental crystalline [Eu(*n*-Pr-BTP)_3_](OTf)_3_ structure and the experimental spectrum.

The B and D absorption resonances are shifted to lower energies for the spectrum calculated with *FEFF* using the DFT optimized structure compared with the spectrum using the experimental structure; the former spectrum has less agreement with the experimental spectrum. This effect is probably due to the 5 pm larger Eu—N bond length in the DFT optimized structure. There is no effect on the pre-edge. For the *FDMNES* calculations on the other hand, the pre-edge of the spectrum using the experimental structure has larger intensity compared with the spectrum using the optimized structure, perhaps due to changes in the electronic density associated with the shorter bond length. B and C exhibit only small differences, suggesting that *FDMNES* treats multiple-scattering effects less accurately than *FEFF*.

**Figure 7:**
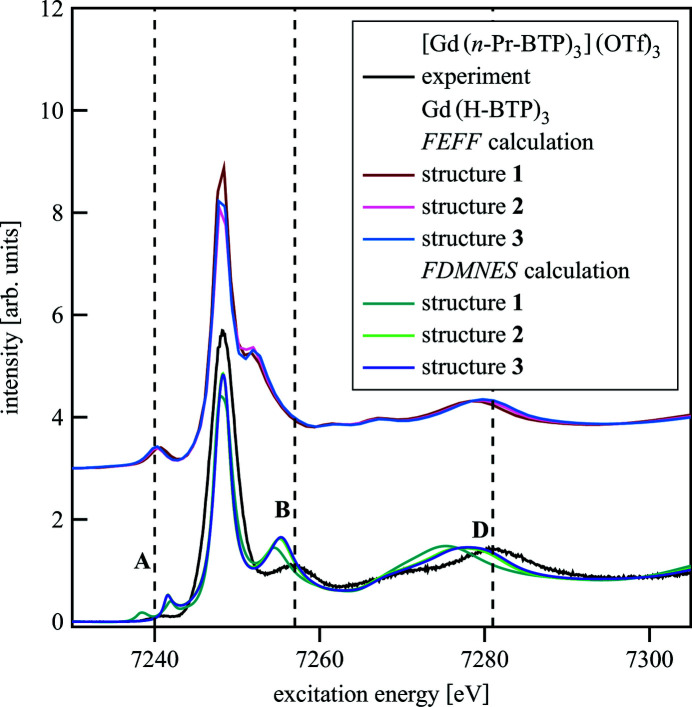
[Gd(*n*-Pr-BTP)_3_](OTf)_3_ experimental spectrum compared with Gd(H-BTP)_3_ spectra calculated with *FEFF* and *FDMNES* for DFT optimized structures with symmetry restrictions (structure **1**), without symmetry restrictions (structure **2**) and without symmetry restrictions and a solvation sphere (structure **3**).

**Figure 8:**
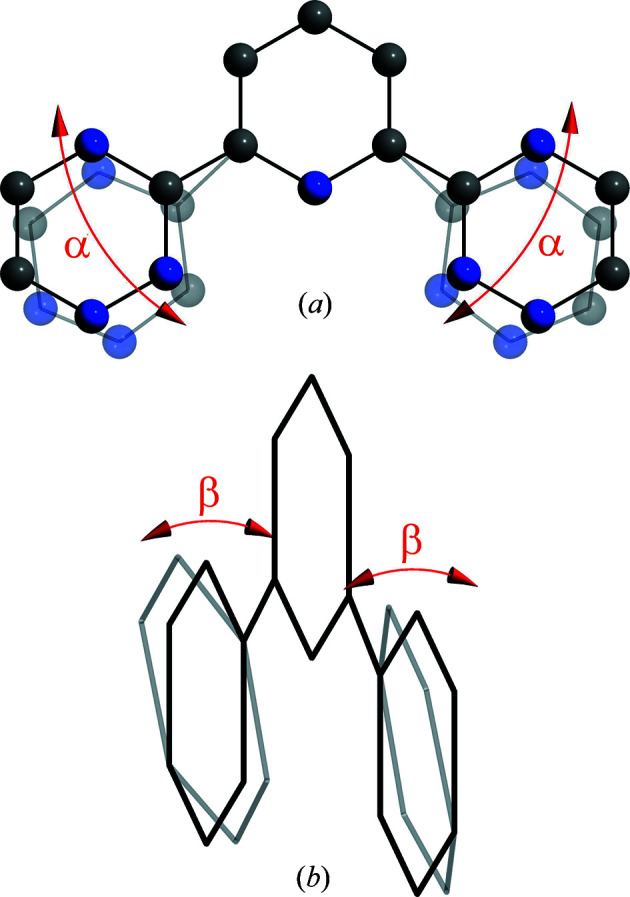
Angles (*a*) and (*b*) describing the in-plane and out-of-plane angles of the triazine rings compared with the pyridine rings.

Fig. 7 ▸ shows the spectra of differently optimized Gd(H-BTP)_3_ structures calculated with *FEFF* and *FDMNES*. Structures were optimized using DFT with symmetry restrictions (structure **1**), without symmetry restrictions (structure **2**) and without symmetry restrictions and a solvation sphere (structure **3**). Gd(H-BTP)_3_ structures were used instead of Eu(H-BTP)_3_ structures because a solvated Eu(H-BTP)_3_ structure was not available. Geometric differences between the structures are summarized in Table 1 ▸, Fig. 8 ▸.

It can be seen that the average Gd—N bond length changes as well as the orientation of the triazine rings with respect to the pyridine ring. In the spectra calculated with *FEFF* a shift to higher energies of B and D can be observed from structure **1** to structure **3** showing that structure **3** is closest to the real structure. The same effect is visible in the spectra for structures **2** and **3** calculated with *FDMNES*. In the spectrum of structure **1**, however, B and D are significantly shifted to lower energies and the pre-edge shows a double structure that is not visible in the other spectra. For the spectra compared here, the spectrum of structure **3** has the best agreement with the experimental spectrum indicating that structure **3** is closer to the real structure than the other two. Further improvements can be expected using higher-level quantum chemical methods, which are not always applicable for large molecules.

**Figure 9:**
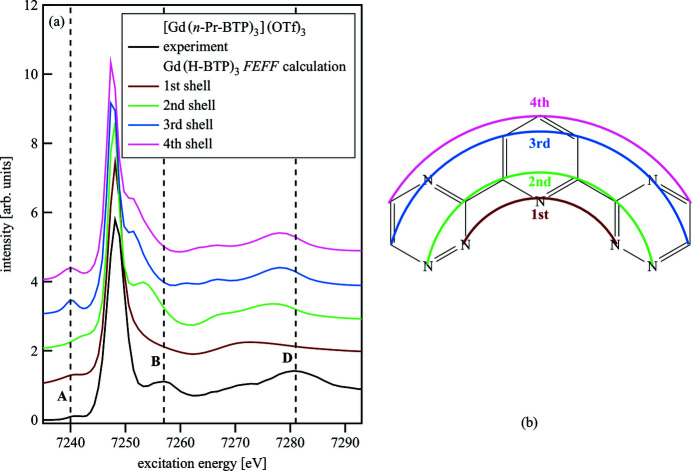
*FEFF* shell-by-shell calculations of Gd(H-BTP)_3_ compared with an experimental [Gd(*n*-Pr-BTP)_3_](OTf)_3_ spectrum (*a*) and the four shells used in the shell-by-shell calculations (*b*).

Fig. 9 ▸(*a*) shows *FEFF* shell-by-shell calculations of Gd(H-BTP)_3_ in a simplified approach to correlate the spectral features with specific groups of atoms surrounding the absorbing atom. The spectra of Gd(H-BTP)_3_ with structure **1** were calculated with SCF and FMS radius increasing from 3 Å to 6 Å in 1 Å steps. A scheme of the used shells is shown in Fig. 9 ▸(*b*). The spectrum only including the contributions from the first shell, *i.e.* the bonding N atoms, is nearly featureless because only a few multiple-scattering paths are available. Adding the non-bonding neighbors of the bonding N atoms to the calculation as a second shell leads to clearly resolved features B and D. When adding additional atoms to the calculation, B is shifted to lower energies, farther away from the experimental energy position, and D is shifted to higher energies, closer to the experimental energy position. These shifts are due to the interference of additional scattering signals and indicate that B is mainly influenced by first- and second-shell atoms while D is related to third- and fourth-shell atoms. The pre-edge A also exhibits small changes probably due to changes in the scattering potentials of the atoms surrounding the Gd.

#### 3.1.3. Comparison between Gd *L*_3_-edge HR-XANES of [Gd(*n*-Pr-BTP)_3_](NO_3_/OTf)_3_ and Gd(NO_3_/OTf)_3_

**Figure 10:**
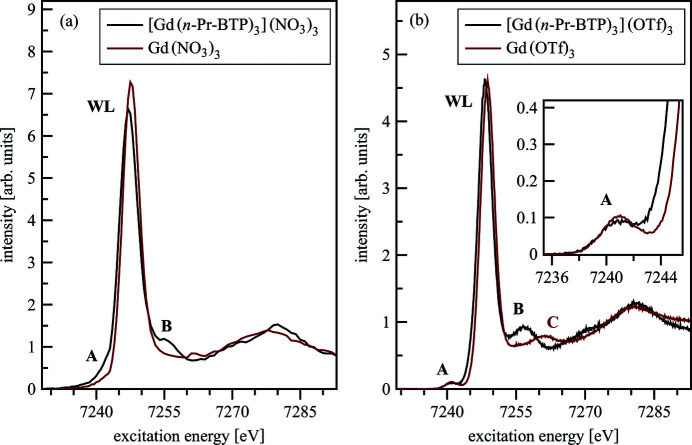
Gd *L*_3_-edge HR-XANES of [Gd(*n*-Pr-BTP)_3_](NO_3_)_3_ and Gd(NO_3_)_3_ (*a*) and [Gd(*n*-Pr-BTP)_3_](OTf)_3_ and Gd(OTf)_3_ (*b*).

Gd *L*_3_-edge HR-XANES spectra of [Gd(*n*-Pr-BTP)_3_](NO_3_)_3_ and Gd(NO_3_)_3_ are shown in Fig. 10 ▸(*a*). The [Gd(*n*-Pr-BTP)_3_](NO_3_)_3_ spectrum is shifted 0.4 eV to lower energies compared with the Gd(NO_3_)_3_ spectrum indicating higher electronic density on the cation in the [Gd(*n*-Pr-BTP)_3_](NO_3_)_3_ complex. This trend is preserved if 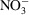 is exchanged with an OTf^−^ anion. The [Gd(*n*-Pr-BTP)_3_](OTf)_3_ and Gd(OTf)_3_ spectra are plotted in Fig. 10 ▸(*b*) showing a 0.5 eV energy shift of [Gd(*n*-Pr-BTP)_3_](OTf)_3_ to lower energies. The energy shifts were validated by ensuring that the measured emission lines coincide in energy position. The additional pre-edge intensity at about 7241 eV (marked with A in Fig. 10 ▸), more noticeable for [Gd(*n*-Pr-BTP)_3_](NO_3_)_3_ compared with Gd(NO_3_)_3_, can be assigned to 2*p*_3/2_ photoelectron transitions to 4*f* and/or 5*d* final states.

**Figure 11:**
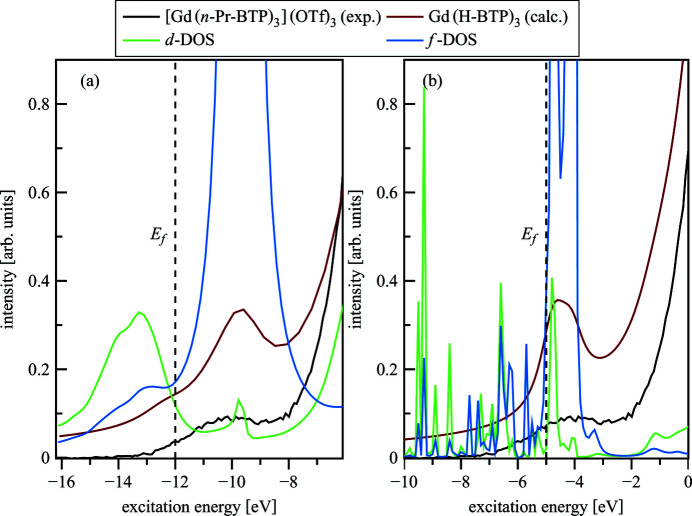
Pre-edge calculated with *FEFF*9.5 (*a*) and *FDMNES* (*b*) for Gd(H-BTP)_3_, including Gd *f* -, *d*-DOS compared with experimental spectra of [Gd(*n*-Pr-BTP)_3_](OTf)_3_.

The origin of the pre-edge feature is revealed by calculations with the *FEFF*9.5 and the *FDMNES* codes. The calculated Gd(H-BTP)_3_ spectrum and the *f*- and *d*-DOS are plotted in Fig. 11 ▸.

The *f*-DOS has high intensity at the energy position of the pre-edge feature, whereas the 5*d* states have minor contributions. This result suggests that this feature arises from electronic transitions to orbitals with major 4*f* and minimal 5*d* Ln participations. Even though the direct bonding partners of Gd change from N in [Gd(*n*-Pr-BTP)_3_](OTf)_3_ to O in Gd(OTf)_3_, hardly any difference between the areas of the pre-edges and WLs is detectable, indicating that the relative electronic populations of the Gd 4*f* and 5*d* states are not significantly influenced by bonding with the *n*-Pr-BTP molecule. Nevertheless, the −0.4 eV relative energy shift of the WL for [Gd(*n*-Pr-BTP)_3_](OTf)_3_ over Gd(OTf)_3_ is a clear indication of better screening of the 2*p* core-hole due to higher electron density on Gd in [Gd(*n*-Pr-BTP)_3_](OTf)_3_ than in Gd(OTf)_3_. The post-edge feature B is at lower energy in [Gd(*n*-Pr-BTP)_3_](OTf)_3_ than the corresponding feature C in Gd(OTf)_3_. In Fig. 10 ▸(*a*) the spectra of [Gd(*n*-Pr-BTP)_3_](NO_3_)_3_ and Gd(NO_3_)_3_ are compared. The same features and energy shifts are noticeable; they are less pronounced due to the different experimental setup resulting in lower energy resolution for [Gd(*n*-Pr-BTP)_3_](NO_3_)_3_ and Gd(NO_3_)_3_. Due to the specific energy resolution of different beamlines/experimental setups and the varying intrinsic broadening of different elements it is not possible to quantitatively compare the areas of the peaks for the different compounds.

#### 3.1.4. Comparison between An *L*_3_-edge HR-XANES of [An(*n*-Pr-BTP)_3_](NO_3_)_3_ and An(NO_3_)_3_

**Figure 12:**
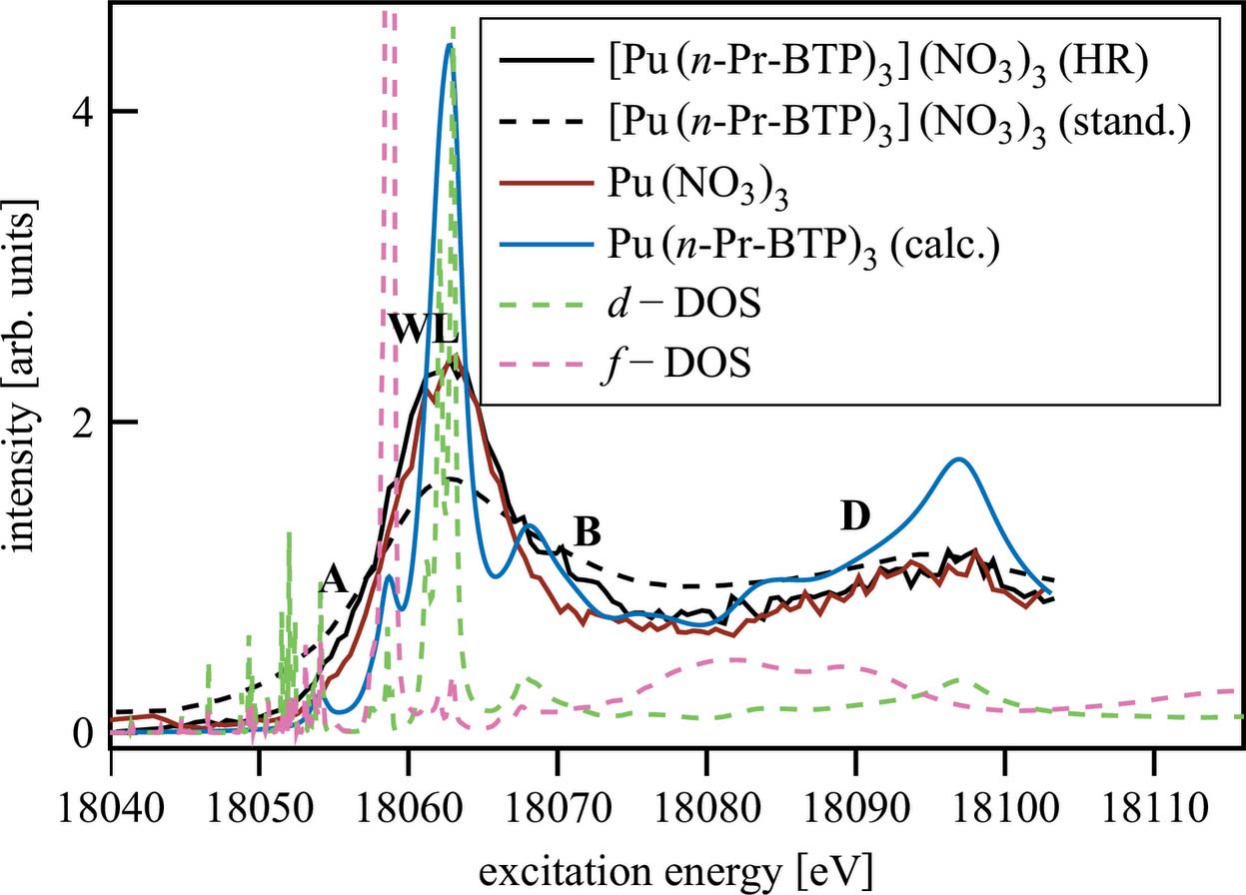
Pu *L*_3_-edge HR-XANES of [Pu(*n*-Pr-BTP)_3_](NO_3_)_3_ and Pu(NO_3_)_3_; Pu *L*_3_-edge XANES of [Pu(*n*-Pr-BTP)_3_](NO_3_)_3_; *FDMNES* calculations of Pu(H-BTP)_3_ XANES and DOS.

[Pu/Am(*n*-Pr-BTP)_3_](NO_3_)_3_ and Pu/Am(NO_3_)_3_ samples have been investigated using Pu/Am *L*_3_-edge HR-XANES. In Fig. 12 ▸ the Pu *L*_3_-edge HR-XANES spectra of Pu(NO_3_)_3_ and [Pu(*n*-Pr-BTP)_3_](NO_3_)_3_ exhibit A and B features that are not resolved in conventional measurements. These spectral features are also characteristic of *L*_3_-edge HR-XANES spectra of isostructural lanthanide complexes reported previously (Prüßmann *et al.*, 2013 ▸) and also here (*cf*. Section 3.1.1). The A and B resonances and the WL in the Pu HR-XANES are less energy resolved due to the higher core-hole lifetime broadening contribution for An (3.3 to 4 eV) compared with Ln (0.8 to 1.6 eV). The WL is broader in the [Pu(*n*-Pr-BTP)_3_](NO_3_)_3_ spectrum compared with the Pu(NO_3_)_3_ spectrum. In addition, feature B is visible only in the [Pu(*n*-Pr-BTP)_3_](NO_3_)_3_ spectrum. Calculation with the *FDMNES* code (Bunău & Joly, 2009 ▸) confirm the presence of pre-edge feature A arising from excitations to a mixture of *d* and *f* states.

**Figure 13:**
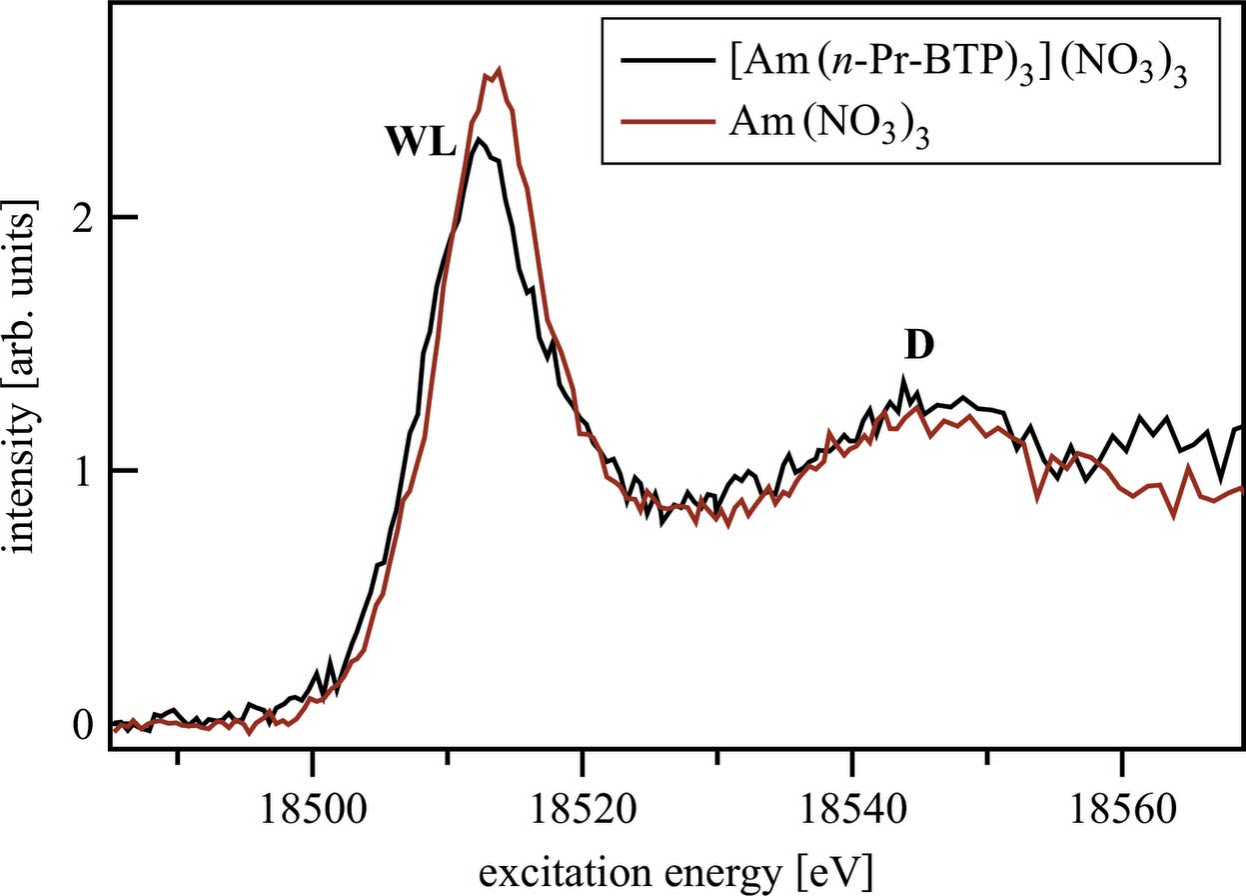
Am *L*_3_-edge HR-XANES of [Am(*n*-Pr-BTP)_3_](NO_3_)_3_ and Am(NO_3_)_3_.

In Fig. 13 ▸, features A and B are not resolved in the Am *L*_3_-edge HR-XANES Am(NO_3_)_3_ and [Am(*n*-Pr-BTP)_3_](NO_3_)_3_ spectra due to the higher core-hole lifetime (Am: 3.87 eV; Pu: 3.74 eV) and experimental broadening (Am: 3.88 eV; Pu: 4.14 eV) for Am compared with Pu. The WL is broader in the [Am(*n*-Pr-BTP)_3_](NO_3_)_3_ spectrum compared with Am(NO_3_)_3_. For both Pu and Am the spectra have similar feature D 35 eV above the WL and the spectra of the complexes are shifted to lower energies (<0.5 eV). This indicates a better screening of the 2*p* core-hole due to higher charge density on the metal in [Pu/Am(*n*-Pr-BTP)_3_](NO_3_)_3_ than in Pu/Am(NO_3_)_3_ similar to the results for the Ln compounds.

**Figure 14:**
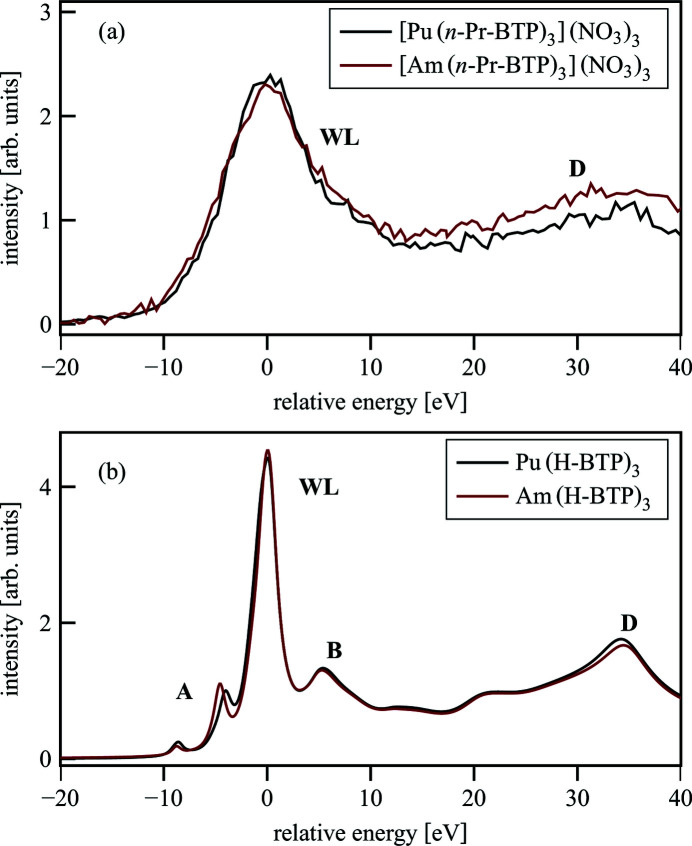
Experimental spectra of [Pu(*n*-Pr-BTP)_3_](NO_3_)_3_ and [Am(*n*-Pr-BTP)_3_](NO_3_)_3_ (*a*) and spectra calculated with *FDMNES* of Pu(H-BTP)_3_ and Am(H-BTP)_3_ (*b*).

The direct comparison of experimental [Pu(*n*-Pr-BTP)_3_](NO_3_)_3_ and [Am(*n*-Pr-BTP)_3_](NO_3_)_3_ spectra [Fig. 14 ▸(*a*)] shows the slightly broader WL of [Am(*n*-Pr-BTP)_3_](NO_3_)_3_. Feature D has different intensity in both spectra due to problematic normalization. In addition, the low signal-to-noise ratio in this range prevents the detection of the expected small variations. The spectra of Pu(H-BTP)_3_ and Am(H-BTP)_3_ calculated with *FDMNES* [Fig. 14 ▸(*b*)] show differences in the pre-edge (A) due to the changing occupation of the 5*f* orbitals (5*f* ^3^ for Pu and 5*f* ^4^ for Am). Feature D in Am(H-BTP)_3_ is shifted to higher energies due to the shorter An–N bond length in Am(H-BTP)_3_ compared with Pu(H-BTP)_3_. Both these effects are not resolved experimentally.

### 3.2. N *K*-edge XANES experiments – feasibility studies

**Figure 15:**
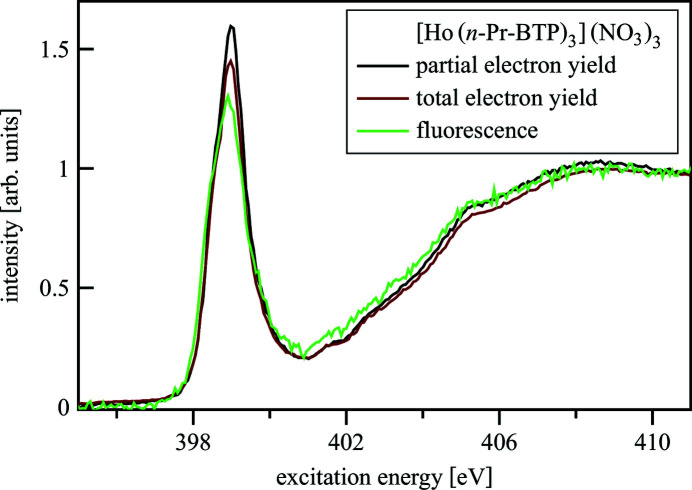
N *K*-edge spectra of [Ho(*n*-Pr-BTP)_3_](NO_3_)_3_ measured in different detection modes.

Fig. 15 ▸ shows N *K*-edge spectra of [Ho(*n*-Pr-BTP)_3_](NO_3_)_3_ measured at the WERA beamline in partial electron yield (PEY), total electron yield (TEY) and fluorescence mode. PEY detection measures electrons emitted from the sample and discriminates between photo- and Auger electrons. This method is surface sensitive up to a depth of 1–2 nm. TEY detection measures all emitted electrons by counting the current necessary to neutralize the sample. Both electron detection methods are susceptible to charge build-up in non-conducting samples like *n*-Pr-BTP and its complexes. They are also not element selective, which is especially problematic for N *K*-edge spectra in the presence of C due to a large non-linear background from the C *K*-edge. Thus, fluorescence detection is generally preferred for materials for N *K*-edge investigations in the presence of C, even though it has worse signal-to-noise ratio and possible self-absorption artifacts compared with the electron detection methods. At the UE52-PGM beamline, however, the fluorescence detector was unavailable for the beamtime, so PEY has been used.

**Figure 16:**
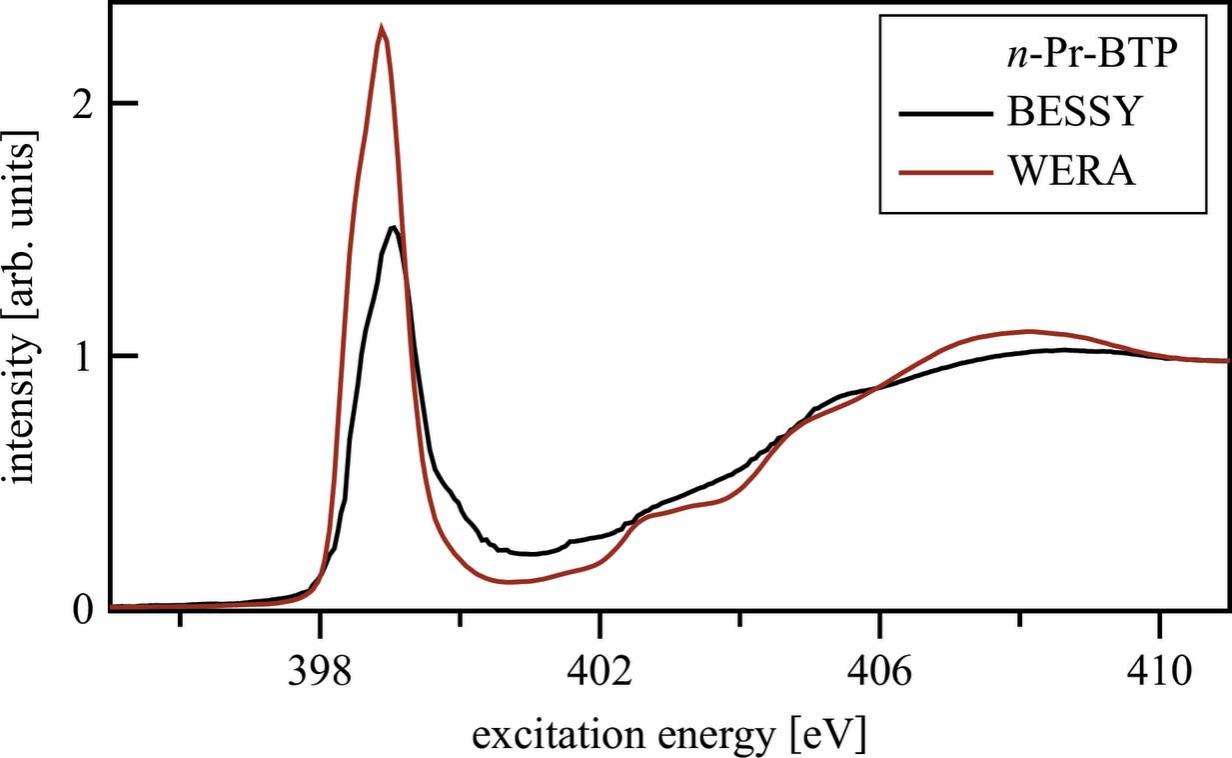
N *K*-edge spectra of *n*-Pr-BTP measured in PEY at the UE52-PGM beamline at BESSY and at the WERA beamline at KARA.

Fig. 16 ▸ shows N *K*-edge spectra of *n*-Pr-BTP measured in PEY at the UE52-PGM beamline at BESSY and at the WERA beamline at KARA. Both spectra have a similar signal-to-noise ratio. The spectrum collected at the UE52-PGM beamline is shifted to higher energies compared with the spectrum collected at the WERA beamline, because at the UE52-PGM beamline no reference spectra could be collected to calibrate the energy scale. In both spectra the FWHM is ∼1 eV and spectral features are equally visible.

**Figure 17:**
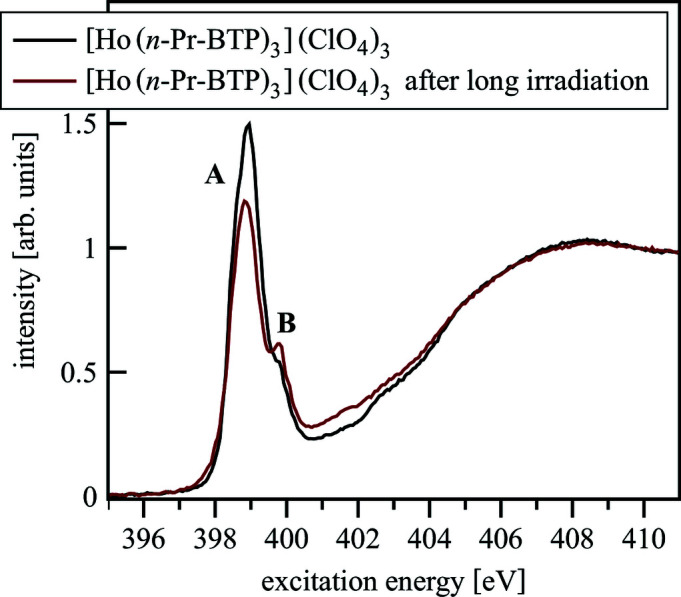
The effects of radiation damage in [Ho(*n*-Pr-BTP)_3_](ClO_4_)_3_ N *K*-edge spectra after irradiation of the samples.

During the measurements at BESSY II, changes of pre-edge features are observed after irradiation of the samples (Fig. 17 ▸). Feature A loses intensity while the intensity of feature B is increased. These effects are attributed to radiation damage, *i.e.* ionization and breaking of bonds by the incident X-ray beam, and were not observed at the WERA beamline (KARA) due to the lower photon flux density impinging on the sample.

**Figure 18:**
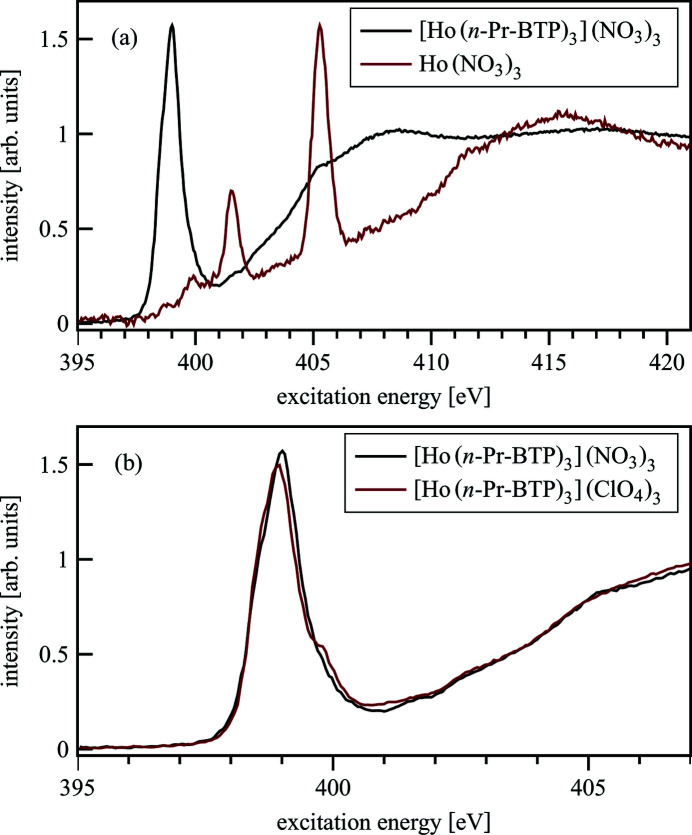
The contribution of 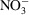 to [Ln(*n*-Pr-BTP)_3_](NO_3_)_3_ N *K*-edge spectra.

Fig. 18 ▸(*a*) shows an N *K*-edge spectrum of Ho(NO_3_)_3_ compared with [Ho(*n*-Pr-BTP)_3_](NO_3_)_3_. The main intensity of the Ho(NO_3_)_3_ spectrum lies at higher energies than the [Ho(*n*-Pr-BTP)_3_](NO_3_)_3_ pre-edge, which is barely influenced. This is confirmed by direct comparison between 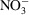 -containing and 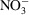 -free {[Ho(*n*-Pr-BTP)_3_](NO_3_)_3_ and [Ho(*n*-Pr-BTP)_3_](ClO_4_)_3_} complexes, see Fig. 18 ▸(*b*).

### 3.3. X-ray Raman spectroscopy – feasibility studies

The process of separation of An from Ln takes place in a liquid phase. However, the N *K*-edge low photon energy (∼400 eV) requires ultra-high-vacuum investigations challenging for liquid samples. First XAS tests with a liquid, constant flow, pump-through cell equipped with 150 nm SiC window (Blum *et al.*, 2009 ▸) at beamline 8.0.1 at the Advanced Light Source (ALS) resulted in fast formation of X-ray induced radiolysis, *i.e.* radiation damage, coloring the *n*-Pr-BTP liquid sample from light orange to dark brown within 2 to 3 minutes. An alternative technique for *K*-edge XANES investigations of low-*Z* elements is X-ray Raman spectroscopy. An incident beam with energies above 10 keV facilitates use of liquid sample cells and double containments necessary for investigations of radioactive materials. The high energy of the X-rays reduces the radiation damage and allows penetration through windows in sample cells made from materials such as Kapton, which has high chemical and X-ray stability and therefore is often used as window material. Disadvantages of the technique are: (1) the low cross section of the process [0.13 cm^2^ g^−1^ inelastic scattering cross section compared with 3 × 10^4^ cm^2^ g^−1^ photo-absorption cross section (Henke *et al.*, 1993 ▸)] requiring at least 3 at% concentration of N atoms in the sample for 2 × 10^12^ photons s^−1^ incident beam, 18 analyzer crystals, (2) the reduced experimental energy resolution when favoring the flux compared with a dedicated soft X-ray beamline leading to broadening of the spectral features. Only a few X-ray Raman spectroscopy measurements of highly concentrated liquid samples are reported in the literature (Bowron *et al.*, 2000 ▸; Bergmann *et al.*, 2002 ▸, 2007 ▸; Näslund *et al.*, 2005 ▸; Juurinen *et al.*, 2013 ▸, 2014 ▸; Wernet *et al.*, 2004 ▸; Pylkkanen *et al.*, 2011 ▸; Sahle *et al.*, 2013 ▸, 2016 ▸; Niskanen *et al.*, 2015 ▸).

**Figure 19:**
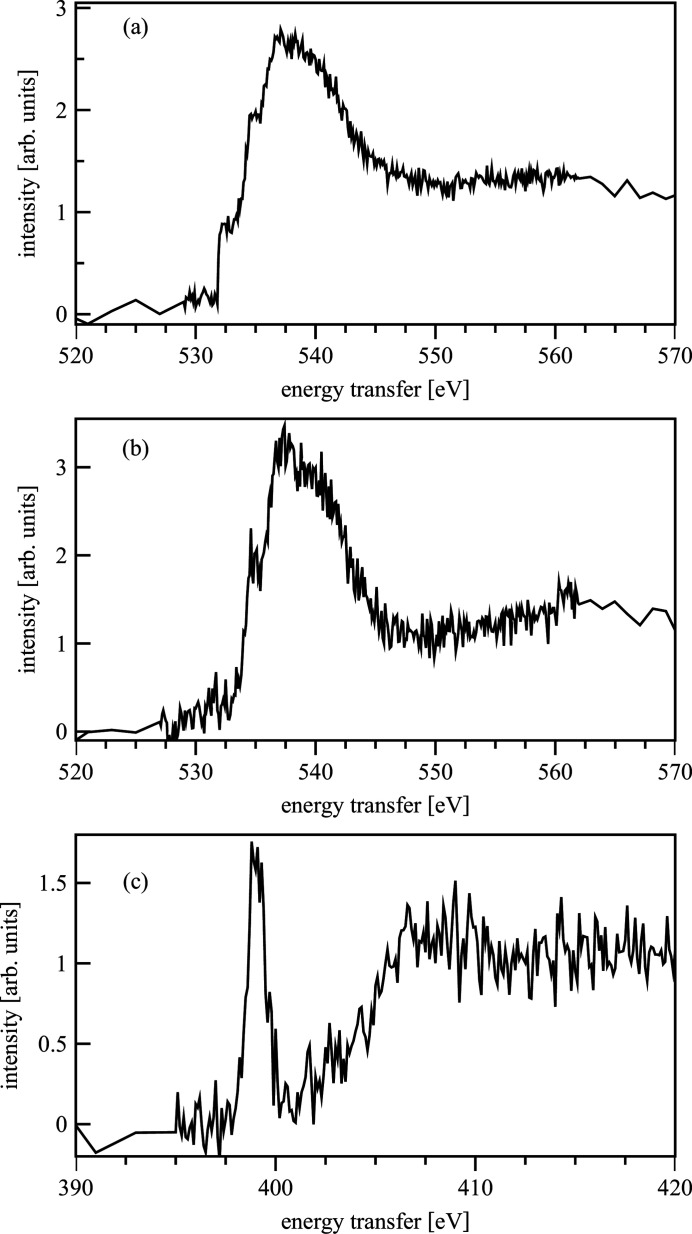
O *K*-edge spectra of iso­propanol (*a*) and water (*b*). N *K*-edge spectrum of *n*-Pr-BTP (*c*).

At the 20-ID beamline at the APS the feasibility studies carried out in November 2012 presented here were the first measurements of liquids performed with the LERIX spectrometer (Fister *et al.*, 2006 ▸); therefore they contributed to the development program of the beamline to extend the range of possible samples. Fig. 19 ▸(*a*) shows the O *K*-edge iso­propanol spectrum after averaging two scans measured 45 minutes per scan. We observed formation of gas bubbles during the measurements probably formed by radiolysis or heating of the iso­propanol. The angle of the cell with respect to the incoming X-ray beam was changed from 45° to 30° to allow the bubbles to escape from the cell without disturbing the measurement. This cell arrangement reduced the number of usable analyzers from 15 to 12. In the O *K*-edge water spectrum [Fig. 19 ▸(*b*)], details for the edge region, similar to those reported for water ice in the literature (Fister *et al.*, 2009 ▸; Zubavichus *et al.*, 2006 ▸), are well distinguishable. The N *K*-edge spectrum of crystalline *n*-Pr-BTP measured for 30 minutes by averaging over 18 detectors is shown in Fig. 19 ▸(*c*). The spectrum has a lower signal-to-noise ratio than the standard XANES spectrum (Fig. 18 ▸). After one scan (30 min) the changed surface color of the sample indicated damage by the beam. A second scan was consistently showing a lower signal. The *n*-Pr-BTP solution revealed no visible color changes after several hours exposure to the beam. However, due to the low concentration no discernible N signal was measured.

**Figure 20:**
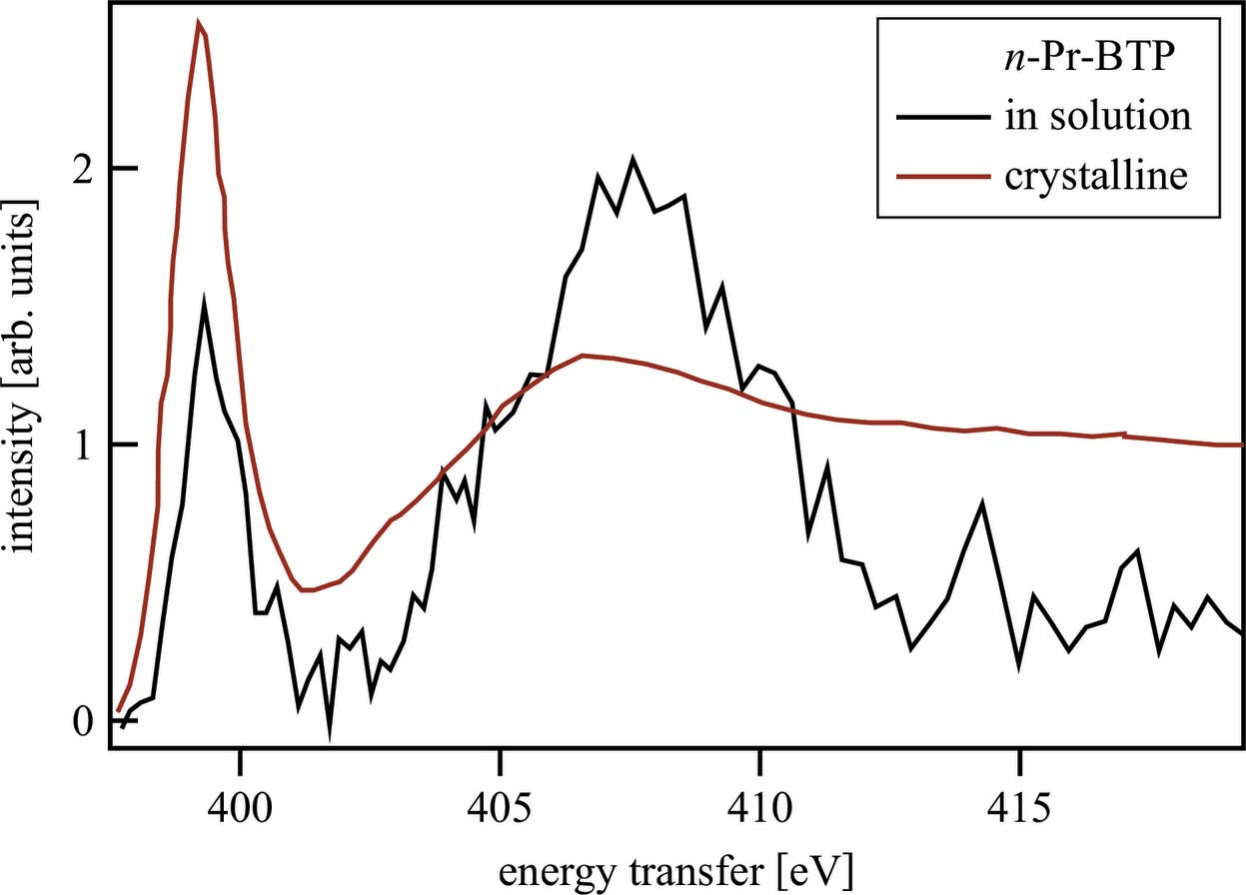
N *K*-edge spectra of *n*-Pr-BTP crystalline and in iso­propanol solution.

At the ID20 beamline at the ESRF (Huotari *et al.*, 2017 ▸) it was possible to measure *n*-Pr-BTP solutions due the higher number of available analyzer crystals and the higher incident photon flux (ID20: 10^14^ photons s^−1^; 20-ID: 10^12^ photons s^−1^). The experiments were performed in November 2014. Fig. 20 ▸ shows the N *K*-edge spectra of *n*-Pr-BTP crystalline and in solution. The crystalline sample has been measured with 48 crystals for 3 h in an evacuated chamber, while the solution sample was measured with 24 crystals for 10 h. The crystalline sample showed similar discoloration as the sample investigated at the 20-ID beamline. A part of the iso­propanol in the solution sample evaporated during the measurements. The pre-edge and WL of the spectra have similar energy positions, but different intensities due to difficulties with the normalization. Even though the signal-to-noise ratio is much worse for the sample in solution, the general structure of the spectrum can be easily recognized and it can be assumed that the structure in solved and crystalline state does not differ significantly. The signal-to-noise ratio of the spectrum of the liquid sample can be improved by utilizing more analyzer crystals and an improved cell design, *e.g.* using a capillary. In addition, the solution should be temperature controlled to reduce evaporation and avoid precipitation of the complex due to temperature difference between preparation lab and beamline.

## 4. Conclusions

The Ln/An *L*_3_-edge HR-XANES technique reveals higher charge density on the metal Ln/An atoms for the [Ln/An(*n*-Pr-BTP)_3_](OTf/NO_3_)_3_ compared with the Ln/An(OTf/NO_3_)_3_ complexes. The high energy resolution allows resolving a pre-edge feature for the [Ln(*n*-Pr-BTP)_3_](OTf)_3_ complexes and it is shown that, as indicated in previous studies (Vitova *et al.*, 2010 ▸, 2013 ▸, 2015 ▸, 2018 ▸; Finkelstein *et al.*, 1992 ▸; Krisch *et al.*, 1995 ▸; Kvashnina *et al.*, 2011 ▸), it originates from electronic transitions to orbitals with predominant 4*f* character. The shape and energy positions of these pre-edges do not change noticeably for the [Ln(*n*-Pr-BTP)_3_](OTf)_3_ and Ln(OTf)_3_ complexes. This strongly suggests that the 4*f* states are localized on the metal atom and do not participate in bonding. The shapes of the pre-edges vary and depend on the number of 4*f* electrons. The 4*f* electrons induce electron–electron interactions leading to a complex structure of the 4*f* states, *i.e.* multiplets. Due to the large core-hole lifetime broadening effects pre-edges are not resolved for the [An(*n*-Pr-BTP)_3_](NO_3_)_3_ complexes. Use of the *L*_β5_ emission lines for measurements of An *L*_3_-edge HR-XANES spectra would lead to reduced broadening effects. The 5*f* states can be also directly probed by An *M*_4,5_-edge HR-XANES and their level of participation in the chemical bond elucidated.

It is demonstrated that the HR-XANES technique allows resolving post-edge features not visible in the conventional spectra. The correlation of their energy positions to specific structural changes, *i.e.* interatomic distances and bonding angles, are revealed with the help of *FEFF* and *FDMNES* XANES quantum chemical calculations and simulations. Both codes are used for detailed analyses of the spectra. Optimized structures with additions of ‘no symmetry restrictions’ and interactions with the solvation sphere improve the agreement between theory and experiment.

The benchmark calculations using various codes and input structures helped to select the most appropriate conditions for the simulations. The input parameters for the *FDMNES* XANES calculations were tested and defined. We found that the best agreement between theory and experiment was achieved using structure **3** [aqueous Gd(H-BTP)_3_], *i.e.* structure **3** is closer to the real structure than the other two.

The benchmark X-ray Raman spectroscopy studies demonstrate the applicability of this novel technique for investigations of liquid samples of partitioning systems at the N *K*-edge and *K*-absorption edges of other low-*Z* elements. No significant differences between the N *K*-edge spectrum for the *n*-Pr-BTP molecule in the solid phase or solved in iso­propanol are found. This result strongly suggests that the results obtained for the solid state complexes and ligands are relevant also for their liquid forms. Specific practical suggestions for further improvement of the experimental set-up are given based on experience gained over several experiments using the state-of-the-art spectrometers installed at the brightest synchrotrons. It is shown that N *K*-edge X-ray Raman spectroscopy investigations of N-donor ligands in solution are in general possible at the ID20 beamline, ESRF. However, the sample setup has to be further improved by using a capillary to allow a high signal-to-noise ratio; temperature-controlled cooling of the sample will reduce the evaporation of the solvent and possible radiation damage.

## Supplementary Material

Tables S1 and S2. DOI: 10.1107/S1600577521012091/yq5003sup1.pdf

## Figures and Tables

**Table 1:** Angles α and β as shown in Fig. 8 ▸ and bond length *R* for differently optimized structures. Structures were optimized using DFT with symmetry restrictions (structure **1**), without symmetry restrictions (structure **2**) and without symmetry restrictions and a solvation sphere (structure **3**)

		α (°)		β (°)		
Structure	Ligand	Left	Right	Left	Right	*R* (pm)
**1**		2.60		1.49		260.7

**2**	1	3.32	3.33	1.73	1.43	258.2
	2	3.35	3.38	1.58	1.44	
	3	3.33	3.32	1.42	1.48	

**3**	1	3.94	3.99	1.90	2.20	256.5
	2	3.96	4.03	1.74	2.04	
	3	3.94	3.99	1.98	2.03	
