# Supplementary material for: Opportunities and challenges of applying advanced X-ray spectroscopy to actinide and lanthanide N-donor ligand systems
Source: J Synchrotron Radiat. 2022 Jan 1;29(Pt 1):53–66. doi: 10.1107/S1600577521012091 (PMC8733980; doi:10.1107/S1600577521012091)
Supplement: Supplementary file 1 [file s-29-00053-sup1.pdf]

JOURNAL OF SYNCHROTRON RADIATION

Volume 29 (2022)

Supporting information for article:

Opportunities and challenges of applying advanced X-ray spectroscopy to actinide and lanthanide N-donor ligand systems

Tim Pruessmann, Peter Nagel, Laura Simonelli, David Batchelor, Robert Gordon, Bernd Schimmelpfennig, Michael Trumm and Tonya Vitova

S1. Example *FEFF* input file

```
*Eu L3 edge in EuBTP3OTf3
EDGE L3
S02 1.0
* pot xsph fms paths genfmt ff2chi
CONTROL 1 1 1 1 1 1
PRINT 1 0 0 0 0 0
EXCHANGE 0 15 −1.5
SCF 4.0
XANES 5.0
FMS 5
MULTIPOLE 2
EGRID
e_grid −20 0 0.2
k_grid last 5 0.05
POTENTIALS
* ipo t Z element l_scmt l_fms stoichiometry
0 63 Eu −1 −1 1
1 16 S −1 −1 4
2 9 F −1 −1 16
3 8 O −1 −1 17
4 7 N −1 −1 21
5 6 C −1 −1 91
ATOMS
*x y z ipo t
0 0 0 0
−0.62329 −1.81917 −1.65159 4
0.70697 0.06468 2.43770 4
−1.57400 1.66066 1.12255 4
−0.44382 1.71040 −1.84110 4
−2.47377 −0.15358 −0.64871 4
2.03844 −1.54171 0.19277 4
1.70133 0.08736 −1.91728 4
1.72800 1.79497 0.61515 4
```

−1.08652 −1.75416 1.54770 4

0.33711 −2.64943 −2.11171 4

−1.07076 2.57031 1.99411 4

2.13545 1.92047 1.86820 5

−1.87595 −2.02233 −2.04284 5

−1.52549 2.51384 −1.77335 4

−2.85333 1.73898 0.79264 5

1.61159 0.96725 2.87559 5

2.14636 −2.37072 1.26361 4

2.90790 −1.65698 −0.79523 5

−0.74092 −1.82747 2.82078 5

0.21827 −0.80395 3.33983 5

0.35894 1.83303 −2.89428 5

−2.89941 −1.07279 −1.53361 5

−3.37234 0.73230 −0.16413 5

1.53520 0.94453 −2.95325 5

2.76966 −0.74692 −1.94519 5

2.21791 2.64543 −0.32129 4

−1.96689 −2.67396 1.05952 4

0.00243 −3.62990 −2.93199 5

−1.87958 3.48830 2.48078 5

−0.89371 −0.53426 −4.56279 3

−1.77166 3.34962 −2.75877 5

3.12132 −3.25715 1.27897 5

−2.81695 0.41663 3.73367 3

3.06190 3.58805 0.05520 5

−2.40465 −3.62104 1.86832 5

−2.29188 −2.99838 −2.85415 4

0.18068 2.67317 −3.91950 4

−3.73006 2.67413 1.22751 4

3.00248 2.86409 2.31508 4

−1.12775 −2.77597 3.68807 4

3.93136 −2.53908 −0.83596 4

2.06148 1.01151 4.19177 5

0.60268 −0.81117 4.67430 5

−4.70502 0.70825 −0.54147 5

−4.22239 −1.16491 −1.95253 5

3.70096 −0.71478 −2.97939 5

2.41678 1.01762 −4.04099 5

−1.35936 −3.81773 −3.31807 5

−3.24622 3.55989 2.07492 5

−0.91675 3.41419 −3.88724 5

4.06596 −3.32989 0.22326 5

3.45116 3.71462 1.41729 5

−1.92566 −3.70277 3.21680 5

1.54490 0.10396 5.10057 5

−5.12823 −0.25557 −1.44264 5

3.51664 0.17837 −4.04242 5

−1.08167 5.30538 0.02587 2

1.32420 −5.44197 −0.16242 3

−2.56593 1.10689 −4.98713 3

1.16292 −4.45091 −3.42690 5

2.18759 −3.96156 3.55176 5

−3.03765 4.14426 −2.60766 5

−1.65403 0.16794 −5.52464 1

0.11641 4.25455 3.89814 5

0.32620 5.59593 −1.49141 2

−2.82786 1.20760 4.93240 1

−1.32056 4.48245 3.47247 5

3.24258 −4.15298 2.48534 5

5.71151 1.14284 −0.26731 5

−1.54005 1.58796 5.43040 3

3.61971 4.51295 −0.98125 5

−3.76644 −4.50901 −0.10124 5

3.36506 4.18222 −2.40435 5

−3.34081 −4.66488 1.32806 5

−0.26799 −5.87706 1.59003 3

−0.35651 6.10810 −0.48527 5

−5.51021 −1.48984 2.30254 5

5.60965 −1.58829 2.12836 3

0.63178 −6.26561 0.62095 1

1.73164 −3.87247 −4.69628 5

5.40835 −0.76758 3.27580 5

−4.23209 3.45860 −3.27719 5

1.51701 6.12313 1.18649 3

−3.64397 −1.54715 −5.25266 2

−3.79070 2.26630 4.95077 3

6.57733 0.04806 −0.90372 5

−1.13503 −6.49945 −1.24386 2

−0.93130 0.59526 −6.64504 3

−1.78379 −4.92609 −4.24774 5

−4.15206 4.63321 2.60574 5

−1.24416 4.26120 −5.08877 5

5.21675 −4.29115 0.24105 5

4.37882 4.80815 1.88369 5

−2.29430 −4.86020 4.16611 5

−2.77087 −1.11749 −6.16301 5

−2.67628 −1.01745 6.29117 2

−1.13503 6.88345 −1.24386 2

−3.42569 0.06624 6.20351 5

−6.71799 −0.52179 2.21463 5

5.28102 −1.51269 4.53903 5

0.63178 7.11729 0.62095 1

2.43982 −4.84779 4.75558 5

−2.09179 −2.18954 −6.57676 2

0.52628 5.27229 4.95693 5

−0.35651 −7.27480 −0.48527 5

−4.65409 −5.68805 −0.55365 5

3.98108 5.23637 −3.32647 5

3.05826 −4.50817 −5.08175 5

5.40835 −5.12814 −0.68684 5

−3.24865 −5.05221 −4.47044 5

4.48432 4.96606 3.36341 5

−5.54295 4.13854 −2.91905 5

1.51701 −7.25977 1.18649 3

−5.54902 4.65441 1.99775 5

−0.11399 4.46119 −6.07634 5

−3.54089 −4.54751 4.87006 5

−4.66258 −0.32943 5.95768 2

−6.90959 0.31520 3.14252 5

−0.26799 7.50584 1.59003 3

2.35009 −0.97180 −7.36213 5

0.93737 5.39468 −5.57411 5

0.32620 −7.78697 −1.49141 2

7.97428 0.02686 −0.29573 5

−3.48390 −0.69539 −7.18591 2

3.16740 2.89414 −6.79343 3

−6.19777 0.79274 −5.06300 5

1.32420 7.94093 −0.16242 3

5.67513 2.07684 5.32677 2

0.69120 2.59263 7.63426 5

−1.08167 −8.07752 0.02587 2

−3.43903 0.61975 7.40806 2

1.29873 −1.90528 −7.86436 5

3.77614 1.10863 7.30443 3

1.70981 −2.57570 7.77790 5

−6.40635 −4.59176 −3.35109 2

−8.19498 2.06727 2.06551 4

0.17462 1.68508 8.54306 5

−8.06038 1.27647 3.12473 5

−6.51670 3.01806 5.02983 3

8.39628 −1.93222 −1.67396 4

7.47225 3.08850 −3.45512 5

6.58339 −0.46781 −5.82052 5

−8.14527 −3.54018 −0.42500 5

8.33565 −2.34005 2.04930 3

4.91238 3.71404 6.44366 2

1.63342 3.50777 8.06053 5

−3.69247 −6.07989 −5.47400 5

8.88012 −1.04646 −0.82655 5

5.46656 6.04808 3.77749 5

−6.24628 −3.74000 −5.28434 2

−6.64160 −0.23495 −6.06655 5

END

S2. Example *FDMNES* input file

```
! La L3−edge in optimized La (H−BTP)_3 structure
Filout
Labtp
Range
−35. 1 80. 5 100 1 200
Edge
L3
Green
SCF
Spinorbite
quadrupole
Eimag
0.1
Density
Radius
8
molecule
1 1 1 90 90 90
57 0 0 0
7 −1.3224387 −1.5224491 −1.8031183
7 −1.3224387 1.5224491 1.8031183
...
1 −6.6325216 0 0
1 3.3162608 −5.7439322 0
1 3.3162608 5.7439322 0
convolution
gamma_max
1
gamma_hole
1
End
```
